# Multispectral 7 Tesla MRI as a potential predictor of dopamine transporter deficiency in Parkinson’s disease

**DOI:** 10.1162/IMAG.a.1241

**Published:** 2026-05-26

**Authors:** Mert Özer, Bernhard Egger, Angelika Mennecke, Armin Nagel, Moritz Zaiss, Frederik Bernd Laun, Arnd Dörfler, Jürgen Winkler, Alexander German

**Affiliations:** Department of Computer Science, Friedrich-Alexander-Universität Erlangen-Nürnberg, Erlangen, Germany; Department of Molecular Neurology, University Hospital Erlangen, Erlangen, Germany; Institute of Neuroradiology, University Hospital Erlangen, Erlangen, Germany; Institute of Radiology, University Hospital Erlangen, Erlangen, Germany

**Keywords:** Parkinson’s disease, dopamine transporter, 7 Tesla MRI, multispectral MRI, contrastive learning

## Abstract

The neuropathological hallmark of Parkinson’s disease (PD) is a progressive degeneration of dopaminergic neurons in the substantia nigra resulting in a reduced striatal dopamine transporter (DaT) concentration. Radioligands can detect degeneration of nigrostriatal projections early in the disease course, but their clinical use is constrained by ionizing radiation exposure and the need for radiotracer production and associated logistics. In this study, we investigate whether a healthy DaT atlas can be predicted directly from native 7 Tesla (7T) multicontrast magnetic resonance imaging (MRI) using a normative atlas as the supervised target for voxel-wise DaT density. Based on these results, we estimate the deviation of sporadic PD patients from the normative DaT atlas. Using this approach, we found that estimated DaT distributions in the putamen differ between PD patients and healthy controls, providing preliminary evidence that high-field advanced multispectral MRI could inform on neurochemical alterations in PD.

## 1 Introduction

PD is a progressive neurodegenerative disorder characterized by the loss of dopamine-producing neurons in the substantia nigra (Dauer & Przedborski, 2003). The ensuing deficit in dopaminergic projections to the striatum, beginning in the dorsal putamen, leads to motor symptoms such as bradykinesia, rigidity, and tremor (Kalia & Lang, 2015; Morrish et al., 1996). The current reference standard for diagnostic imaging of PD depends on the extraordinary biomolecular specificity and picomolar sensitivity of radiotracer scans in nuclear medicine (Catana et al., 2008). A dopamine transporter (DaT) scan is a nuclear imaging method that uses the radioligand ^123^I-FP-CIT to reveal the loss of dopaminergic synaptic terminals in the putamen, whereas conventional MRI appears normal (Heim et al., 2017; Parkinson Study Group, 2000). However, nuclear medicine imaging of the human brain is limited by exposure to ionizing radiation and the need for radiopharmaceutical production and administration, which in the case of ^123^I-FP-CIT involves a delay of several hours between tracer injection and image acquisition, and DaT measurements are influenced by medication-dependent changes in transporter availability, constraining broad screening and repeated assessments. Broad screening for PD is of interest because neurodegeneration is a process in the basal ganglia that is known to begin years to decades before the onset of motor symptoms, highlighting the urgent need for the development of non-invasive and reliable biomarkers for early detection and disease monitoring (Ferrer et al., 2012). In addition, novel PD imaging markers are attractive to complement clinical examination, which faces challenges in distinguishing PD from atypical parkinsonian syndromes such as multiple system atrophy or progressive supranuclear palsy with overlapping clinical features in particular at the onset of disease (He et al., 2018).

Dopaminergic synaptic terminals represent a small fraction of the volume in the putamen, but exert a pronounced regulatory effect on its function in motor control, which is responsible for the prototypical motor symptoms observed in PD patients (Albin et al., 1989; DeLong, 1990; Rice et al., 2011). Some studies using magnetic resonance (MR) spectroscopy, which is among the most sensitive tools of molecular MRI, have indicated small alterations in the putaminal myo-inositol, total creatine and total N-acetylaspartate content in PD, while others detected no change (Abe et al., 2000; Davie et al., 1995; Mazuel et al., 2016; Watanabe et al., 2004). It is, therefore, an open question whether the neurochemical alterations in PD sufficiently affect the physical tissue properties that can be acquired using MRI.

MRI has been a central tool in clinical neurology, most commonly through T1-weighted, T2-weighted, and fluid-attenuated inversion recovery (FLAIR) imaging to visualize gross anatomy and lesions. Yet, as primarily structural and morphological contrasts, these clinical sequences do not provide direct cellular or molecular information, which makes them semi-quantitative, low in sensitivity, and difficult to interpret clinically (van Zijl & Yadav, 2011). Recent advances in MRI have begun to bridge this gap. For example, ultra-high-field 7T MRI improves spatial resolution and sensitivity to subtle tissue changes; q-space trajectory imaging (QTI) captures microstructural properties of tissue such as neurite orientation and heterogeneity, and chemical exchange saturation transfer (CEST) offers molecular sensitivity to metabolites and pH, making it one of the few MR techniques directly linked to neurochemistry (Jones et al., 2018; Ladd et al., 2018; Westin et al., 2016). We have demonstrated that multi-contrast MRI is able to capture discriminative voxel-wise tissue signatures, while Hu et al. (2023) reported that diffusion and perfusion metrics reflect the genetic, metabolic, and invasive heterogeneity of glioblastoma (German et al., 2021). Extending these insights, recent diffusion MRI gradient analyses have revealed microstructural alterations in the posterior putamen in PD, and CEST MRI in a PD mouse model (Drori et al., 2025; Shmuely et al., 2025).

These findings motivate further research into the possibility of predicting nuclear medicine reference imaging results from multispectral MRI data, which would both aid in the biological interpretation of high-dimensional MR properties and preselect candidates for specific radiotracers in clinical routine. In this work, we utilize high-field 7T MRI with advanced contrasts, including QTI, CEST, quantitative susceptibility mapping (QSM), and standard clinical contrasts, to learn a high-resolution voxel-wise normative DaT distribution (Haacke et al., 2015). We employ a two-stage learning framework: first, a contrastive learning approach (an unsupervised machine learning method) is used to derive a subject- and coordinate-invariant embedding that enhances the discriminative power of multispectral voxels; second, a supervised feed-forward multi-layer perceptron (MLP) is trained to predict the DaT atlas from these embeddings. Application of our pipeline to PD patients predicts significantly reduced putaminal DaT density, in accordance with the known pattern of dopaminergic synaptic loss in PD.

## 2 Datasets

### 2.1 DaT PET atlas

**Fig. 1.**
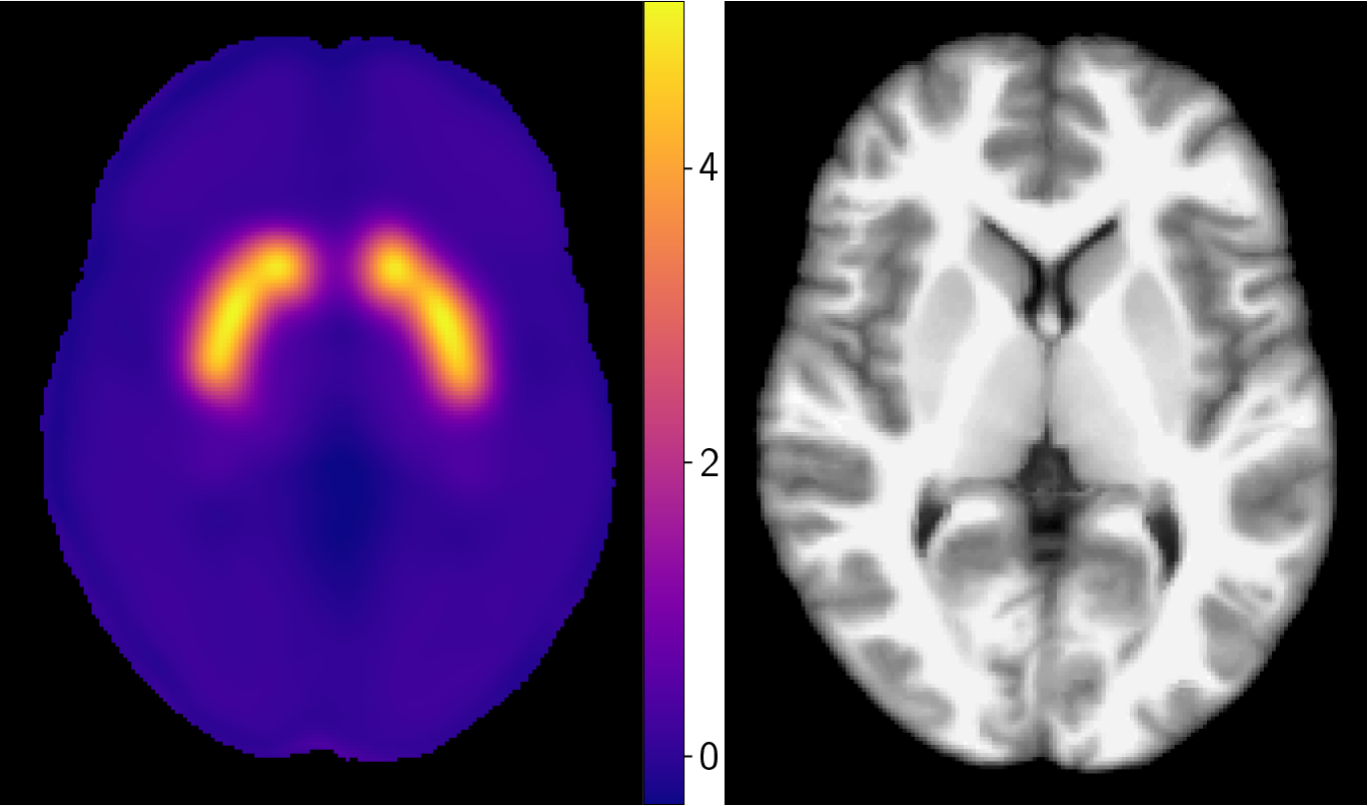
The healthy DaT atlas reported by Sasaki et al. (2012), averaged over 10 healthy men with mean age 28.1 years. Right is the corresponding MNI-152 structural image (Postelnicu et al., 2009).

We use the healthy DaT atlas (Fig. 1) reported by Sasaki et al. (2012), who quantified DaT in healthy adults with 90-minute dynamic positron emission tomography (PET) using the radioligand ^18^F-FE-PE2I. Ten healthy men (mean age 28.1 years) were scanned; uptake followed known DaT densities of regions such as the striatum or the midbrain, and binding potentials with cerebellum as the reference region were stable with 60-minute data, indicating minimal radiometabolite bias. This provides a high-contrast, regionally specific DaT map (high in the putamen/caudate; moderate in the midbrain; low in the thalamus; minimal in the cerebellum) suitable as a healthy reference. For further details, we refer to the study by Sasaki et al. (2012) and Hansen et al. (2022).

### 2.2 Multispectral MRI

The multispectral MRI dataset was acquired previously by us using a 7T MRI scanner (Magnetom Terra, Siemens Healthineers AG, Erlangen) (German et al., 2021). In total, 38 subjects were scanned. The cohort comprises 26 healthy controls (HC), including 8 young men (mean age 24.9 years, SD 1.45, range 22-27) and 18 older adults (mean age 57.8 years, SD 6.17, range 48–69; 4 women and 12 men), as well as 12 patients with sporadic PD (mean age 58.4 years, SD 7.86, range 48–73; 2 women and 10 men), with a mean disease duration of 2.27 years (SD 1.42) and a mean levodopa equivalent daily dose (LEDD) of 238 mg/day (SD 243.3).

PD diagnoses were established in the specialized movement disorders outpatient clinic of University Hospital Erlangen by experienced neurologists according to the Movement Disorder Society clinical diagnostic criteria for Parkinson’s disease (Postuma et al., 2015). The diagnostic work-up included neurological history and examination, assessment of dopaminergic treatment response, review of exclusion criteria/red flags, structural brain MRI to exclude secondary causes of parkinsonism, and longitudinal clinical follow-up. Dopaminergic imaging was available in 5 of 10 patients as part of routine clinical care when clinically indicated, but was not required for study inclusion because PD diagnosis remains primarily clinical.

All participants provided written informed consent to participate in the study, which was approved by the local ethics committee. The inclusion of both healthy and diseased participants provided a wide range of tissue and pathology signatures, and high-resolution imaging was performed to ensure precise voxel-level analysis.

Each voxel was characterized by a rich multispectral profile of 351 features. This spectrum combined both derived parametric maps and raw signal intensities from complementary MRI contrasts. From diffusion acquisitions, QTI yielded 15 parametric maps capturing anisotropy, diffusivity, and microscopic orientation dispersion, while the raw diffusion signals from b-tensor encoding were included across linear, planar, and spherical schemes (210 values). CEST imaging contributed four Lorentzian-fitted amplitudes corresponding to magnetization transfer, nuclear Overhauser effect (NOE), and amide/amine peaks, as well as 112 raw z-spectrum samples acquired at varying nominal frequency offsets, without voxel-wise correction for $B_{0}$. Standard anatomical contrasts included high-resolution Magnetization Prepared – RApid Gradient Echo (MPRAGE), while susceptibility-based methods (QSM and susceptibility-weighted imaging (SWI)) were acquired at 0.6 mm isotropic resolution. All acquisitions were performed as a slab centered on the basal ganglia and midbrain, except MPRAGE, ensuring coverage of dopamine-rich projection regions while keeping acquisition times feasible at 7T. All contrasts of a subject were rigidly registered to its MPRAGE space to ensure voxel-wise correspondence across modalities. This pipeline produced a dataset in which every voxel carried a high-dimensional multispectral fingerprint. Further details of the PD patients are presented in Supplementary Table S1, and for other details of multispectral image acquisition, we refer to German et al. (2021).

## 3 Methods

### 3.1 Preprocessing

All MRI data were preprocessed to achieve voxel-wise correspondence across subjects while minimizing spurious sources of variability. To establish a common space across participants, the full-brain MPRAGE images were reconstructed and non-linearly registered to MNI space using the FreeSurfer combined volumetric and surface-based (CVS) registration algorithm (Fischl, 2012; Postelnicu et al., 2009). The resulting deformation fields were applied to the multispectral slab acquisitions. This established a correspondence between all voxel signatures across subjects.

**Fig. 2.**
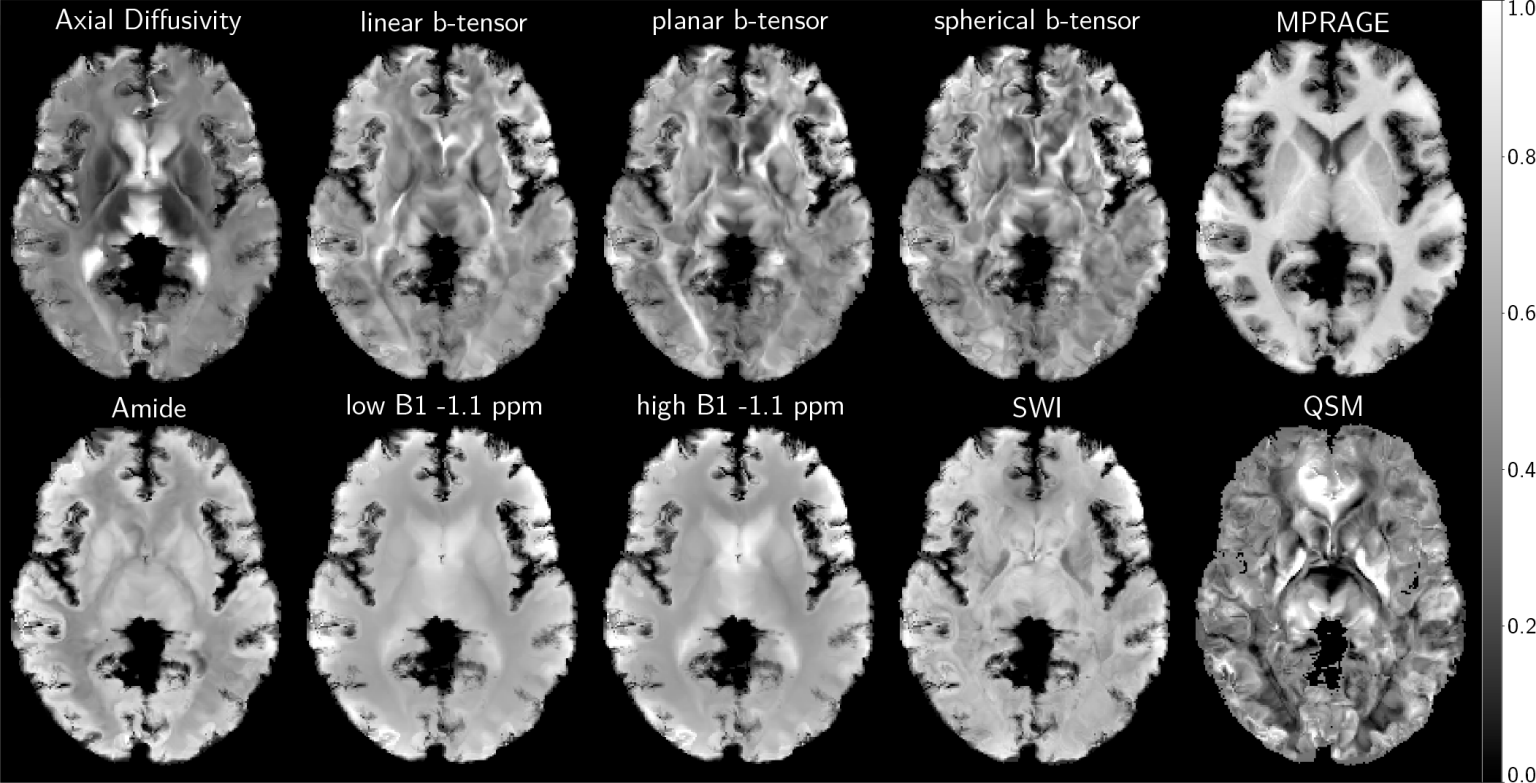
A selection of polynomial-detrended non-linear MNI space registered multispectral MRI contrasts.

To reduce trivial location-specific signal biases, such as $B_{0}$ or $B_{1}$ inhomogeneities, we fit a fourth-order polynomial function to the voxel coordinates and subtracted the fit from the data. This procedure effectively removed the smooth spatial trends linked to anatomical position, leaving only residual variance reflecting tissue properties. The residual values were then z-scored per subject, to retain within-subject variance structure while aligning scales across participants, and subsequent analyses were performed on these standardized residuals. A selection of contrasts from the dataset is presented in Figure 2.

Finally, five HC and two PD patients were excluded due to data quality, and two linear b-tensor indices were removed from all subjects. After these steps, the dataset comprised normalized, high-quality 349-dimensional voxel signatures from 21 HCs and 10 PD patients. The HC group included 6 younger controls (mean age 24.8 years, SD 1.67, range 22–27) and 15 older controls (mean age 57.3 years, SD 6.15, range 48–69). PD patients had a mean age of 58.7 years (SD 8.15, range 48–73), a mean disease duration of 2.5 years (SD 1.46), and a mean LEDD of 249.6 mg/day (SD 249.8). This final cohort was used for all downstream embedding and predictive modeling.

### 3.2 Contrastive embedder

The goal of the embedding step is learning a voxel-wise representation that reflects tissue information while being free of coordinate and subject-ID biases. These unique biases per subject are present in each scan due to magnetic field inhomogeneities, revealing a voxel’s location in 3D or the subject it belongs to. Consequently, any learning-based method applied to multispectral MRI can collapse to the trivial solution, memorization of the subject-specific biases rather than the actual task. We, therefore, pretrain an encoder with contrastive learning and adversarial removal of residual non-tissue cues. We use PyTorch as the main machine learning framework in this study (Paszke et al., 2019).

For each 349-D voxel i, we generate two independently augmented views $i^{\left(1\right)}=t_{1}\left(i\right)$ and $i^{\left(2\right)}=t_{2}\left(i\right)$, where $t_{1},t_{2}∼T$ , forming a positive pair; embeddings from all other voxels in the batch are treated as negative samples. All transforms from the augmentation set T operate on the multispectral feature vector, simulating scanner/batch effects while preserving tissue identity: (i) global multiplicative/additive intensity shifts ($B_{1}$/baseline drift), (ii) smooth low-rank per-channel gain (coil/spectral-profile variation), (iii) monotone gamma-like warps (non-linear readout differences), (iv) additive Gaussian noise, and (v) random feature dropout. Augmentations are sampled independently for the two views and the strength is ramped up through the training.

An MLP encoder maps the augmented 349-D input vectors to $L^{2}$-normalized 16-dimensional embeddings, r. The encoder is composed of 4 hidden layers of size 2,048; each followed by ReLU activation, layer normalization, and dropout with 0.3 chance (Ba et al., 2016; Ioffe & Szegedy, 2015; Nair & Hinton, 2010; Srivastava et al., 2014).

A temperature-scaled InfoNCE loss aligns positives (augmented views) and pushes apart all other samples in a batch:

$${ℒ}_{\text{Contrastive}}=\frac{1}{2N}\sum_{i=1}^{2N}\left[-\text{log}\frac{\text{exp}\left({\text{sim}}_{ii^{\prime }}/\;\tau \right)}{\sum_{\begin{array}{c} k=1\\ k\neq i \end{array}}^{2N}\text{exp}\left({\text{sim}}_{ik}/\;\tau \right)}\right],$$

where 2N is the number of augmented voxels, $sim_{ii^{\prime }}$ and $sim_{ik}$ denote the cosine similarity of the positive and negative samples (van den Oord et al., 2018). The temperature parameter, τ, adjusts the granularity of the learned embeddings, which is set to 0.07 in this study.

To further eliminate the non-linear biases in the embeddings, two adversarial heads, small MLPs, are attached via gradient-reversal layers (GRL). A coordinate head predicts discretized MNI coordinates and a subject head predicts subject identity. GRL inverts the gradients at the encoder, forcing it to remove spatial and subject information. Both of the heads share the same architectural specifications: three 512-dimensional hidden layers with each followed by a layer normalization, ReLU activation and a dropout of 0.3 chance.

Each coordinate axis (x,y,z) is normalized to [0,1] and discretized into 16 empirical-quantile bins, yielding a discrete coordinate label $c_{i}^{a}\in \left\{1,\ldots ,16\right\}$ for voxel i along axis a∈{x,y,z}. The coordinate head outputs logits for each axis, while the subject head outputs logits for each subject. A standard cross-entropy loss is used for both, with the coordinate loss averaged across the three spatial axes:

$${ℒ}_{\text{Coordinate}}=\frac{1}{2N}\sum_{i=1}^{2N}\frac{1}{3}\sum_{a\in \left\{x,y,z\right\}}\left(-\text{log}p\left({\hat{c}}_{i}^{a}=c_{i}^{a}\right)\right),$$

$${ℒ}_{\text{Subject-ID}}=\frac{1}{2N}\sum_{i=1}^{2N}\left(-\text{log}p\left({\hat{s}}_{i}=s_{i}\right)\right).$$

Here, 2N denotes the total number of augmented voxels in a contrastive batch, $s_{i}\in \left\{1,\ldots ,C_{\text{subject}}\right\}$ is the subject identity label, ${\hat{c}}_{i}^{a}$ and ${\hat{s}}_{i}$ denote the predicted coordinate-bin and subject labels, respectively, and p(⋅) denotes the softmax-normalized class probability output of the corresponding adversarial head.

**Fig. 3.**
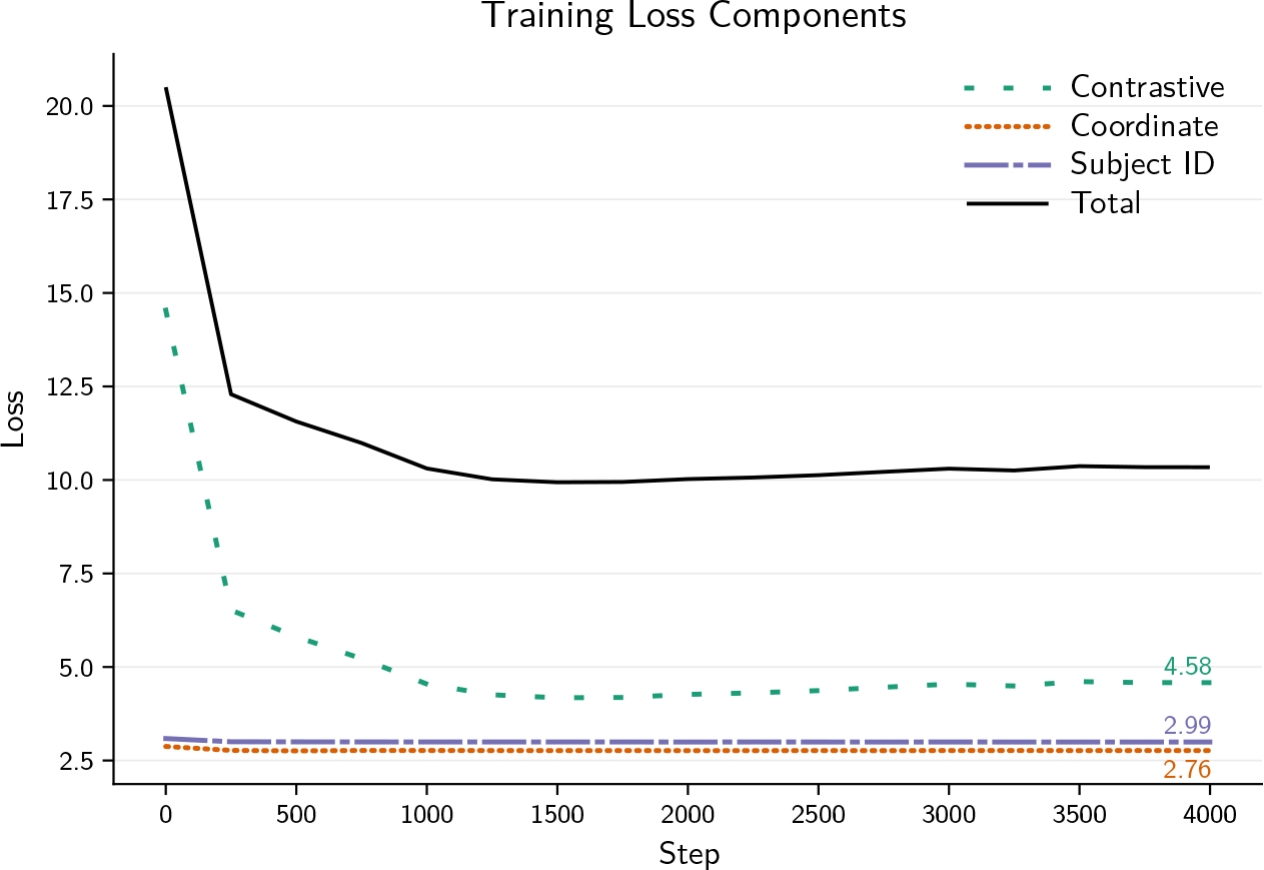
Components of our contrastive embedder loss. Due to gradient reversal at the adversarial heads, even though they are updated by the optimizer, the loss of the coordinate and subject head stays at the chance level throughout the training, showing that the encoder is successful at hiding biases while minimizing the contrastive loss. The slight increase in contrastive loss toward the end of training is caused by the gradual increase in augmentation strength.

The total loss for the embedder becomes:

$$ℒ={ℒ}_{\text{Contrastive}}+w_{\text{c}}{ℒ}_{\text{Coordinate}}+w_{\text{s}}{ℒ}_{\text{Subject-ID}},$$

with $w_{\text{c}}=20$ and $w_{\text{s}}=20$ in practice. High emphasis on the adversarial losses ensures the importance of the bias-free representations as shown in Figure 3. We train for 4,000 steps using AdamW (learning rate $1\times 10^{-4}$ , weight decay $10^{-2}$ ), batch size of 16,384, with automatic mixed precision (Loshchilov & Hutter, 2019). After training, adversarial heads are discarded and the encoder is frozen for downstream use. An example embedded test HC is presented in Supplementary Figure S1.

### 3.3 DaT predictor

We train a feed-forward regressor to map embeddings to voxel-wise DaT values. The regressor shares the exact architecture of the adversarial heads, which is known to be incapable of recovering any bias of the data from the embedding step. For each subject, the input $r\in {\mathbb{R}}^{16}$ is the frozen embedding produced by the contrastive encoder; the regression target y∈ℝ is the corresponding DaT value sampled from the single normative atlas (standardized to zero mean and unit variance).

We minimize mean-squared error (MSE) over voxels in each mini-batch:

$${ℒ}_{\text{MSE}}=\frac{1}{B}\sum_{i=1}^{B}∥f_{\phi }\left(r_{i}\right)-y_{i}{∥}_{2}^{2},$$

where $f_{\phi }$ denotes the MLP and B is the batch size.

**Fig. 4.**
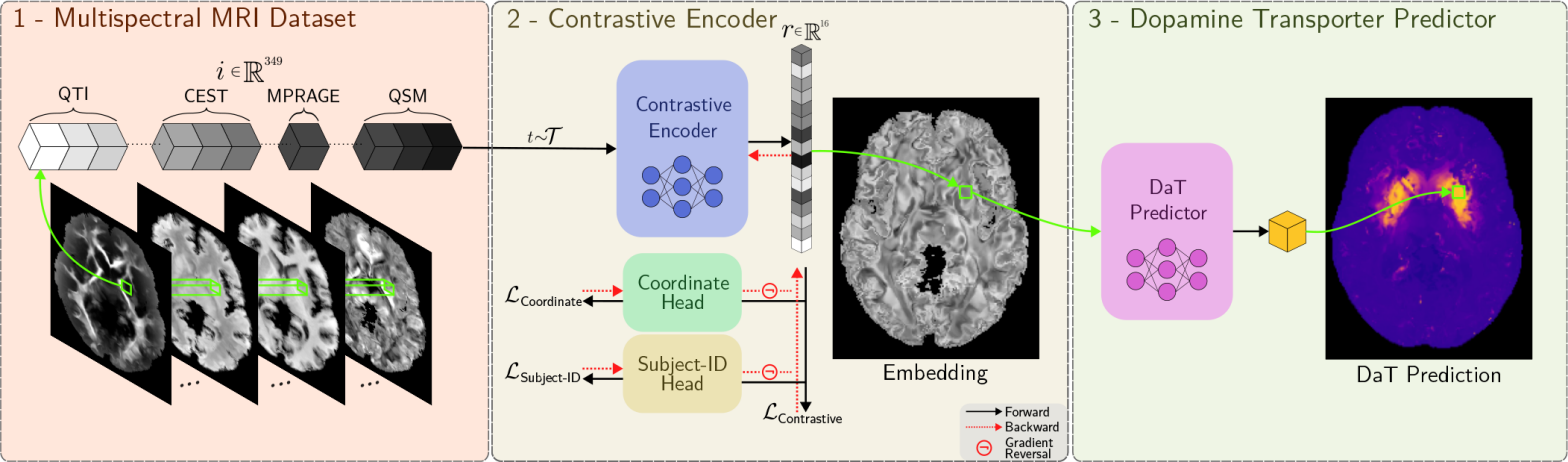
The complete pipeline of DaT prediction from multispectral MRI. Preprocessed 349-dimensional voxels i are augmented as t(i), where t∼T and T denotes the set of augmentation transforms, and then fed to a contrastive encoder. Gradients from the coordinate and subject heads are negated at the encoder, forcing it to learn an embedding r that is free of spatial and subject-specific biases. Finally, an MLP predicts subject-level DaT scans from the bias-free embeddings.

We use AdamW with learning rate $1\times 10^{-3}$ , weight decay $1\times 10^{-2}$ , batch size 4,096 , train for 30 epochs with automatic mixed precision. Additionally, a cosine-annealing scheduler is also employed (Loshchilov & Hutter, 2017). This step completes our pipeline as shown in Figure 4.

### 3.4 Cross-subject validation

To prevent information leakage, both the embedder and the predictor are trained within a leave-one-HC-out cross-validation scheme. In each fold, one HC and all PD subjects are excluded from the whole training pipeline. The embedder is trained on the remaining 20 HCs and then used to embed the entire dataset. Subsequently, the predictor is trained on embeddings from the same 20 HCs, and predictions for that fold are made on the held-out HC and all PD subjects. This setup provides subject-level predictions while ensuring that no information from the test HC or the PD patients influences training.

### 3.5 Evaluation

Evaluation was carried out at FreeSurfer segmented regions and at the group level to assess both prediction accuracy and biological validity. First, subject-level performance was measured by predicting DaT values across all voxels. Second, we restricted analysis to striatal regions of interest (ROI), specifically the caudate and putamen, to test whether predictions in dopamine-rich projection regions diverge in PD.

Each subject’s ROI prediction distributions were further summarized as ROI means for statistical tests by using the subject’s FreeSurfer segmentation. Group differences were assessed with two-sided Welch’s *t*-tests, and *q*-values were controlled at FDR <0.05 using the Benjamini–Hochberg procedure across all ROIs. We report exact q-values, Hedges’ g, and 95% confidence intervals (CI).

To assess diagnostic utility, we computed receiver operating characteristic (ROC) curves based on the ROI mean and median predictions, treating HCs as the negative class and PD patients as the positive class (Pedregosa et al., 2011). We report the area under the ROC curve (AUC), sensitivity, specificity, and balanced accuracy at the optimal threshold determined by the Youden index (Sensitivity+Specificity−1 ).

## 4 Results

### 4.1 Voxel-wise prediction

**Fig. 5.**
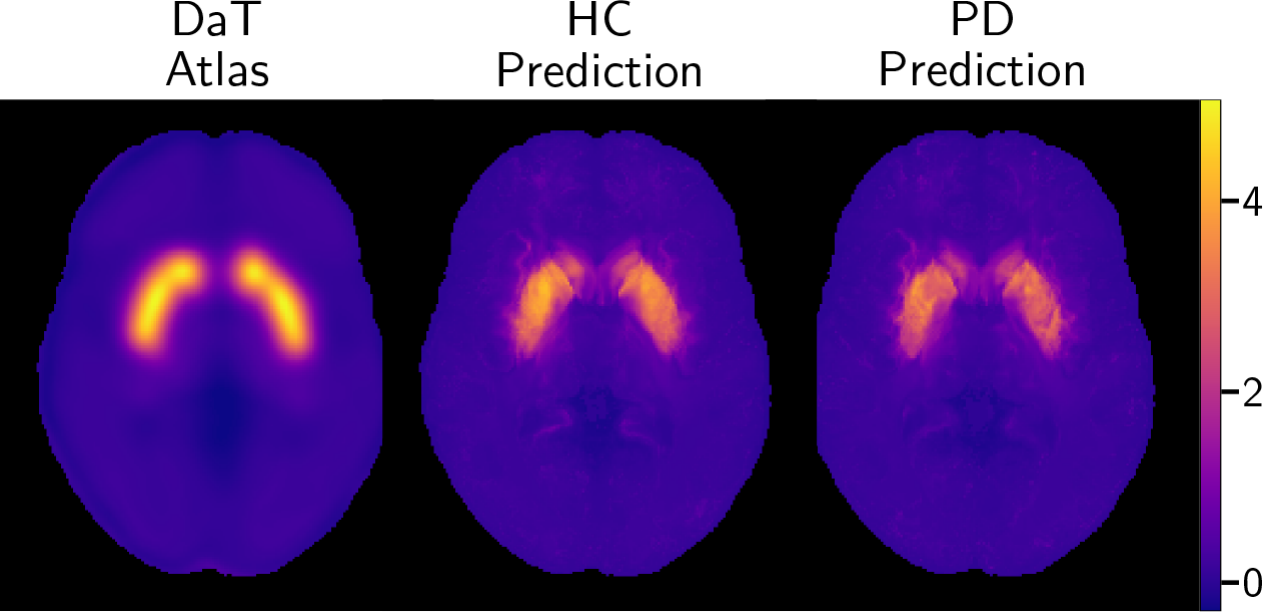
Mean DaT predictions of healthy and PD subjects over each cross-validation fold. PD patients are also averaged across folds. An apparent loss of signal in PD patients is present in visual examination.

**Fig. 6.**
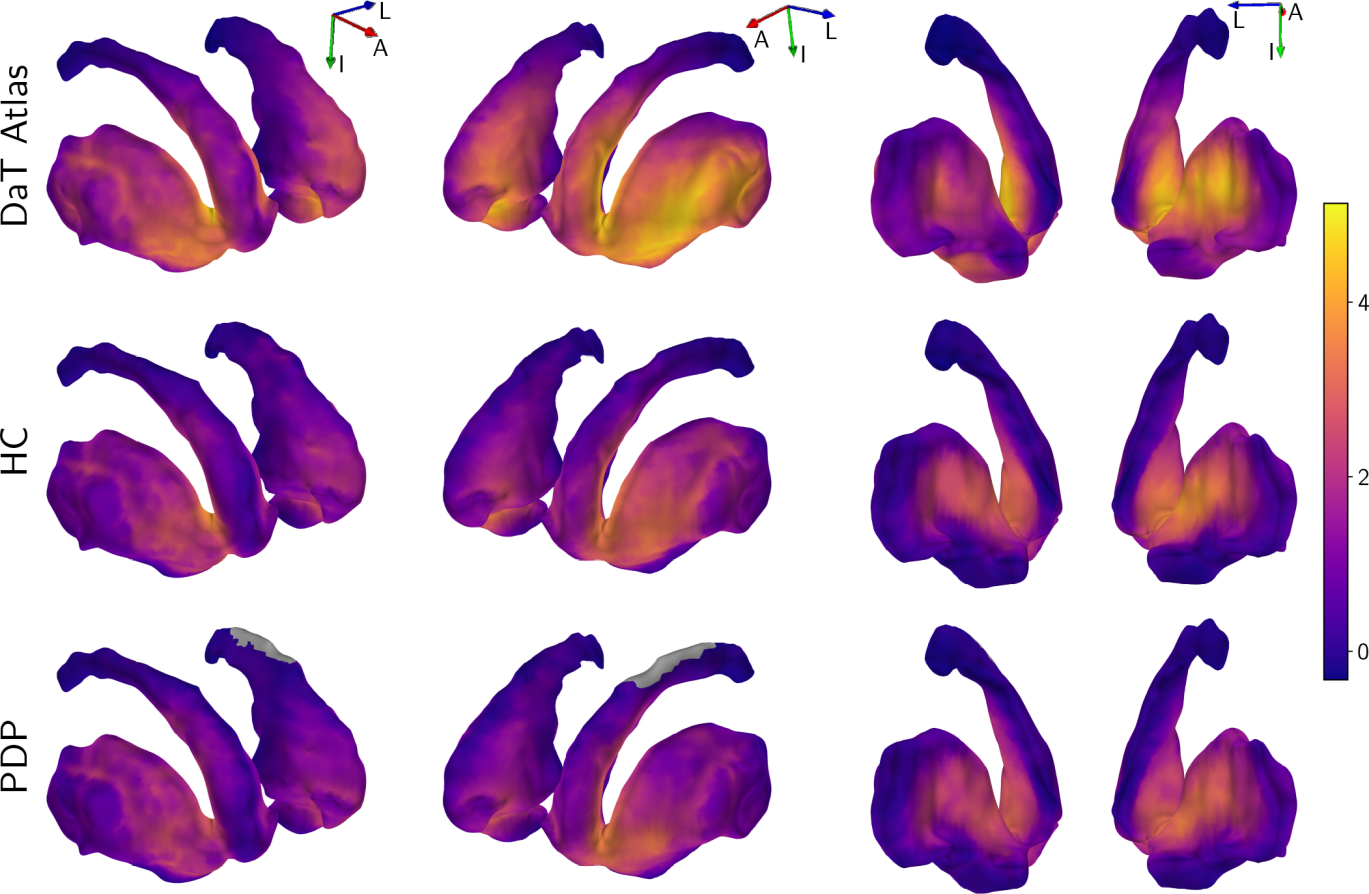
Group-level mean striatum maximum intensity projections of the DaT predictions along the mesh normals. Predictions follow the distribution pattern visible in the target DaT atlas, with pronounced signal intensity in the left putamen. Signal reduction is most pronounced in the putamen, consistent with the known pattern of reduced dopaminergic input in PD. The gray region in the left anterior caudate is not present in the acquisition due to the angulation of the imaging slab. Orientation markers indicate the inferior (I), anterior (A), and left (L) directions.

The trained model successfully translated multispectral MRI inputs from subjects who were not part of the training data into predicted DaT binding potential maps that closely resemble the normative ground truth. Visual inspection of the group-level averages shown in Figure 5 reveals the characteristic high-intensity “comma” shape of the striatum, matching the spatial distribution of the DaT atlas. In contrast, the PD average map demonstrates a visible reduction in signal intensity, particularly localized to the putaminal region. This loss of signal is consistent when examining individual subject predictions provided in the Supplementary Material (Supplementary Fig. S2), where PD patients show fragmented or dimmed predicted putaminal signals compared with the uptake seen in HCs. The spatial distributions of the predictions are visualized in Figure 6, which displays maximum intensity projections of the striatum. The DaT atlas shows the highest signal in the left ventral striatum, which is recapitulated by average predictions for HC and PD groups with visible attenuation in PD.

### 4.2 Quantitative ROI analysis

**Fig. 7.**
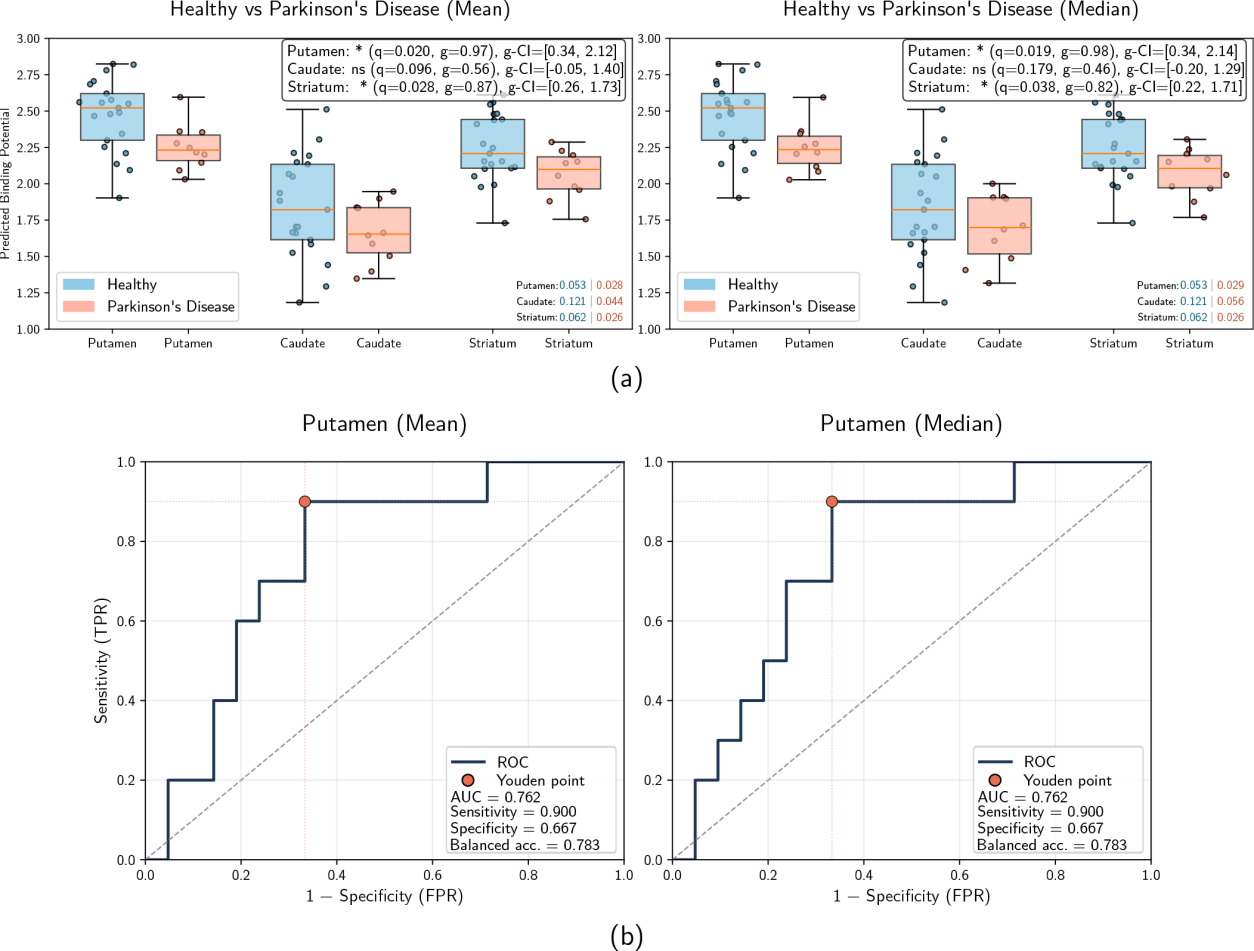
(a) Mean and median DaT predictions for the striatal regions. Blue box plots denote healthy subjects, red box plots denote PD subjects. The legends report FDR-adjusted q, Hedges’ g, and 95% CIs. *, significance level q<0.05 . “ns”, not significant. Numbers in the bottom right indicate the variance within the respective groups for the region. (b) Mean and median ROC curves for PD classification in the putamen. Both curves are flipped and balanced accuracy is defined as the mean of sensitivity and specificity, that is, (Sensitivity+Specificity)/2 . The optimal threshold corresponds to a binding potential of 2.36 for both curves.

To quantify these observations, predicted DaT concentrations were summarized within striatal ROIs defined by FreeSurfer segmentation. Figure 7 presents the boxplots of the atlas-derived predicted binding potentials for the putamen, caudate, and whole striatum, along with ROC curves for individual diagnostic performance in the putamen. For HC, each fold contributes the prediction for one subject and is displayed individually. For PD patients, predictions are averaged across folds (left panels: mean, right panels: median across runs). Additionally, Figure 5 shows voxel-wise group-level averages; individual voxel-wise predictions for qualitative inspection are provided in the Supplementary Material. A statistically significant reduction in predicted DaT binding was observed in the putamen of PD patients compared with HCs. This finding was robust across aggregation methods for the PD cohort, yielding significant results using both the mean and the median across cross-validation runs (mean aggregation: q=0.020 , g=0.97 , 95% CI [0.34, 2.14]; median aggregation: q=0.019 , g=0.98 , 95% CI [0.35, 2.16]). In contrast, differences in the caudate nucleus were not statistically significant after FDR correction (mean: q=0.096 , g=0.56 ; median: q=0.179 , g=0.46 ), suggesting the model correctly identifies the putamen as the primary site of dopaminergic deficit. When combined, the whole striatum also showed significant group differences (mean: q=0.028 , g=0.87 , 95% CI [0.24, 1.74]; median: q=0.038 , g=0.82 , 95% CI [0.19, 1.72]). Both ROC curves, based on mean and median aggregations, yield identical performance for the putamen: AUC of 0.761 , sensitivity of 0.9 , specificity of 0.667 , and balanced accuracy of 0.783 .

### 4.3 Ablation studies

**Fig. 8.**
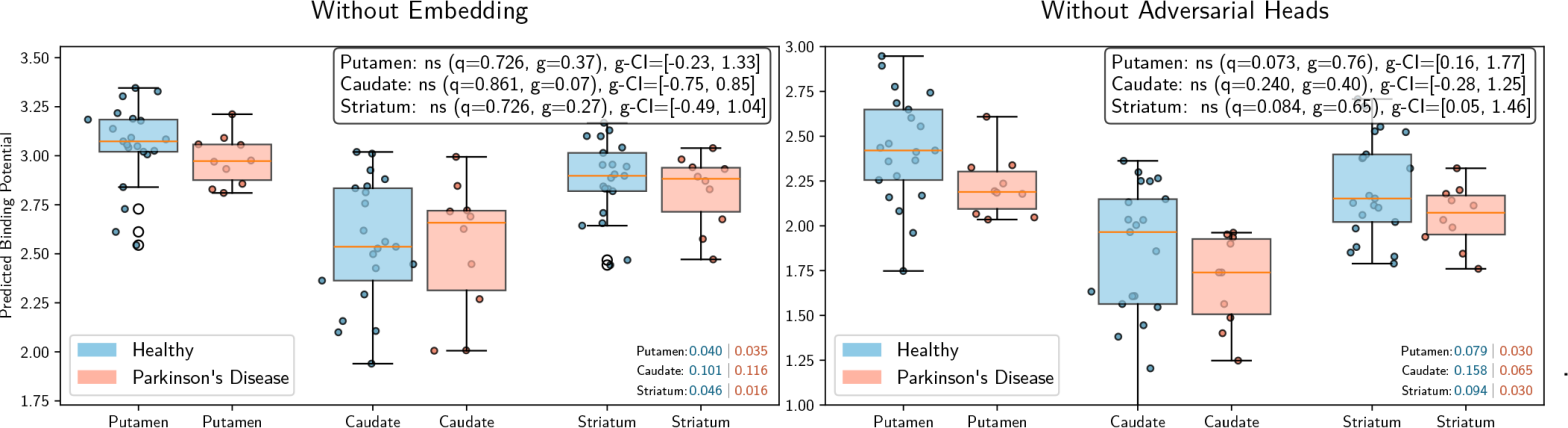
Ablation tests of the pipeline. Left: Mean prediction results without the embedding step show no group difference. Right: Mean prediction results without the adversarial heads. HC vs. PD differentiation increases, however, not reaching statistical significance.

We validated the necessity of our pipeline components—specifically the contrastive embedding and adversarial heads—through ablation experiments shown in Figure 8. Training a regressor directly on the raw multispectral features resulted in no significant differentiation between groups (q=0.726 for putamen), indicating that the embedding step is crucial. Removing the adversarial heads (which suppress subject and coordinate bias) improved differentiation slightly but failed to reach statistical significance (q=0.073 for putamen), confirming that explicit bias removal is required to isolate the disease-specific signal.

### 4.4 Robustness to age bias

**Fig. 9.**
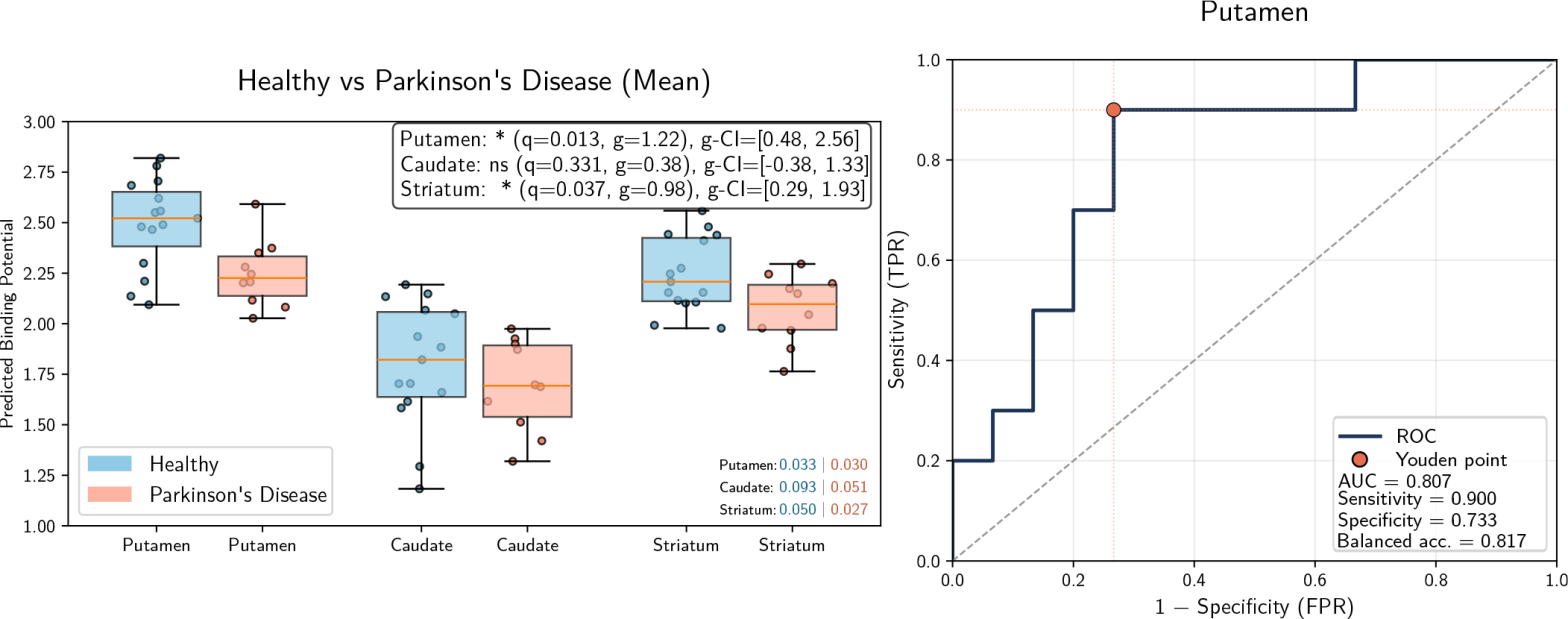
Group comparison after excluding younger HCs. Left: The difference between older HCs (mean age 57.3 years, SD 6.15, range 48–69; n=15 ) and PD patients (mean age 58.7 years, SD 8.15, range 48–73; n=10 ) remains significant, confirming robustness to age-related bias. Right: Diagnostic performance based on mean ROI predictions in the putamen, showing an increased AUC of 0.807 compared with the full-cohort results.

Given the age difference between the overall HC and PD cohorts, we performed a control analysis by excluding younger HC subjects and retaining only those age matched to the PD group. As illustrated in Figure 9, the reduction in putaminal DaT binding remained statistically significant even within this older sub-cohort (q=0.013 , Hedges’ g=1.22 ). In addition, a higher individual diagnostic performance is achieved within this cohort with an AUC of 0.807 . This confirms that the observed DaT depletion is driven by the PD-associated pathology rather than age-related confounds. An age-predicted binding potential correlation analysis is presented in Supplementary Figure S3.

## 5 Discussion

This study demonstrates that an embedding learned from 7T multispectral MRI—explicitly regularized to suppress coordinate and subject identity—supports voxel-wise prediction of a normative DaT distribution and reveals PD-related deviations at the regional level.

While our model targets a normative atlas rather than subject-specific ground truth, the spatial pattern of the predicted deficit of pronounced reduction in the putamen with relative preservation of the caudate mirrors the topography of dopaminergic loss established by nuclear medicine approaches. Quantitative PET and SPECT studies report that putaminal binding is reduced by 50−70% in early-to-moderate PD, whereas caudate uptake is relatively spared (30% reduction) until later stages (Asenbaum et al., 1998; Ikeda et al., 2019). Our results (Hedges’ g≈0.97 in putamen vs. non-significant changes in caudate) capture this anteroposterior gradient, lending biological plausibility to the model. The method achieved moderate diagnostic performance in the putamen (AUC =0.761 ), with high sensitivity (0.90 ) but lower specificity (0.667 ), resulting in a balanced accuracy of 0.783 . Non-significant caudate effects might reflect milder pathology outside the posterior putamen. The ability of multispectral MRI to recover this spatial phenotype from an averaged atlas provides a proof-of-principle that MRI contrasts contain information related to the organization of dopaminergic terminals. This observation is of hypothesis-generating nature and suggests that multispectral MRI may capture aspects of the striatal dopaminergic system, similar to neuromelanin-related contrast or iron-induced susceptibility changes that have been shown to correlate with striatal DaT binding (Isaias et al., 2016; Park et al., 2023).

A body of research has focused on the loss of the dorsolateral nigral hyperintensity (the swallow-tail sign) on iron-sensitive SWI in PD. Meta-analyses report high diagnostic accuracy for the swallow-tail sign, with pooled sensitivities of 94% and specificities of 90% (Chau et al., 2020; Mahlknecht et al., 2017). However, the swallow-tail sign is absent in up to 19% of healthy older adults, leading to false positive diagnosis, and its visualization is dependent on both field strength and motion artifacts (Mahlknecht et al., 2017; Schmidt et al., 2017). Similarly, neuromelanin-sensitive MRI (NM-MRI) has demonstrated separation of PD and controls in small cohorts by quantifying substantia nigra volume loss (Lakhani et al., 2024). Yet, NM-MRI lacks standardized acquisition protocols across vendors, and meta-analyses indicate that heterogeneity in segmentation methods impacts reproducibility (Cho et al., 2021). Other quantitative metrics, such as QSM and diffusion tensor imaging (DTI), reveal group-level differences in iron content and microstructural parameters but suffer from substantial overlap between patients and controls, limiting their utility for individual diagnosis (Pyatigorskaya et al., 2014; Ravanfar et al., 2021).

Beyond these substantia nigra-focused biomarkers, several MRI-based biomarkers targeting the striatum or putamen have also been proposed for the diagnostic work-up in PD, but their biological interpretation and diagnostic performance remain heterogeneous. Early diffusion MRI work at 1.5T reported that mean diffusivity and FA increase in the posterior putamen over a 6-year disease progression period, although FA changes remain inconsistent across studies (Chan et al., 2016; Zhang & Burock, 2020). Susceptibility-based approaches have also been investigated. A study combining QSM with DaT-SPECT detected a moderate inverse association between putaminal susceptibility and dopaminergic deficit (r=−0.478 ), indicating that MRI explains approximately 23% of the variance in dopaminergic loss. This likely reflects the fact that susceptibility measurements capture multiple biological and chemical measures—including iron, myelin, microstructure, and vasculature—rather than exclusively dopaminergic terminals (Uchida et al., 2020). Similar limitations apply to microstructural MRI approaches. Gradient analysis of multiparametric quantitative MRI (qMRI) has shown that asymmetry of microstructural gradients in the posterior putamen correlates with DaT-SPECT asymmetry and motor symptoms ($R^{2}\approx 0.25$ ), but the underlying contrasts (R1, R2*, macromolecular tissue volume) reflect again mixed tissue properties (Drori et al., 2022). A 3T susceptibility study using tractography-based striatal parcellation reported limited diagnostic performance for conventional anatomical regions such as the putamen (AUC ≈0.62 ), with improved classification (AUC ≈0.80 ) only after focusing on the caudal motor striatum (Alushaj et al., 2023). Deep learning approaches combining QSM and T1-weighted MRI have achieved higher classification performance (AUC ≈0.90 internally and 0.845 on external validation), but these models are trained directly on diagnostic labels and, therefore, detect MRI patterns associated with PD rather than estimating dopaminergic synaptic loss (Wang et al., 2023). A regression approach by Bae et al. predicted striatal DaT-SPECT uptake from susceptibility-weighted imaging of nigrosome-1 in the substantia nigra and reported a strong correlation (r≈0.74 ) (Bae et al., 2023). However, this strategy relies on imaging the substantia nigra and requires manual localization of nigrosome-1. In contrast, our approach analyzes multispectral MRI directly within the striatum, where nigral dopaminergic projections are present and DaT-SPECT imaging is obtained. Rather than indirectly inferring terminal degeneration from nigral pathology, our model estimates MRI patterns that resemble the spatial organization of dopaminergic terminals and quantifies the deviation of individual subjects from a healthy distribution. A recent multiparametric qMRI study has similarly reported altered spatial gradients in the posterior putamen, including increased water fraction and reduced R2* gradients in PD, but linking these signal changes to biological alterations remains challenging (Drori et al., 2025). In contrast, our framework does not rely on interpreting individual MRI contrasts but instead learns MRI features that approximate the spatial pattern of dopaminergic terminals derived from molecular imaging, allowing disease-related effects to be interpreted as deviations from healthy/physiological dopaminergic targets of the striatum.

MRI is not currently recommended as a standalone tool for establishing the diagnosis of PD based on clinical guidelines, its primary role remaining the exclusion of secondary etiologies such as vascular parkinsonism and tumors (National Institute for Health and Care Excellence, 2017). MRI biomarkers still lack biological specificity compared with molecular imaging; for instance, iron accumulation and volume loss may occur in atypical parkinsonism or aging, whereas DaT-SPECT provides a direct readout of the dopaminergic nerve terminal integrity (Zarkali et al., 2024). Consequently, functional dopaminergic imaging is recommended over structural MRI (National Institute for Health and Care Excellence, 2017). Our approach attempts to bridge this gap by mapping the rich, albeit non-specific, information available in 7T multispectral MRI directly to the biologically specific domain of DaT.

Prediction of molecular distributions from structural or functional imaging is related to the field of synthetic PET. Studies in Alzheimer’s disease have successfully utilized deep learning to impute amyloid and tau PET status from MRI (Lee et al., 2024; Ten Kate et al., 2018). In the context of PD, Bae et al. (2023) demonstrated that a deep learning regressor trained on nigrosome MRI could predict striatal DaT-SPECT-specific binding ratios. Our work extends this paradigm by using a voxel-wise normative atlas prediction from high-dimensional 7T MRI.

Given the complexity of the high-dimensional input space and processing pipeline components to avoid memorization of confounding variables, we performed an evaluation of our architectural choices. A saliency analysis of our pipeline is provided in the Supplementary Material.

The two ablation experiments in Figure 8 validate the pipeline components. The embedding step provides the largest benefit, substantially enhancing subtle tissue representations within the individual voxels. Adversarial heads then remove residual non-linear biases after preprocessing, yielding the final performance gains.

A valid concern regarding these results is the impact of age distribution within the dataset. To address this, we repeated the statistical analysis after excluding younger HCs, retaining only the older HCs who represent the majority of the cohort. As shown in Figure 9, the effect remained significant (q=0.013 , g=1.22 ), even with higher diagnostic performance (AUC =0.807 ), indicating that the observed difference is not related to age-related bias. To further examine potential confounds, we assessed correlations between predicted binding potential and both putamen volume and age. These analyses were included to evaluate whether the predictions might simply reflect regional atrophy or age-related effects; however, neither correlation reached significance (Supplementary Fig. S3). Together, the findings of the present study suggest that the observed group differences are unlikely to be driven primarily by such biases.

From a methodological standpoint, mean PD predictions were aggregated across folds (mean or median) because each cross-validation split yields a different out-of-fold estimate for PD patients, whereas HC predictions are displayed per fold. We, therefore, provide the per-fold PD predictions compared with the test HC-cohort prediction in Supplementary Material (Supplementary Fig. S4).

Beyond these methodological considerations, several limitations of the present work should be noted. The study relies on 7T MRI, which presents its own set of challenges (Welton et al., 2023). Although ultra-high field imaging provides increased signal-to-noise and contrast-to-noise ratios that enable higher spatial resolution, it is associated with greater $B_{0}$ and $B_{1}$ field inhomogeneities, which required explicit correction in this study. In addition, the limited availability of 7T MRI systems restricts broader applicability. Another limitation is the relatively long acquisition time of the multispectral imaging protocol (∼1 hour), which increases susceptibility to motion artifacts.

Furthermore, the spatial distribution of DaT predictions across the striatum is shown in Figure 6. The figure highlights a clear dependence on the normative DaT atlas, such as higher predicted signal in the lateral left putamen and lower signal in the medial left putamen, indicating that the model recovers spatial patterns from multispectral MRI that resemble the distribution observed in a healthy young DaT atlas. Consequently, the predictions should be interpreted as deviations from this molecular reference rather than as a subject-level quantification of dopaminergic binding. Without paired PET/SPECT measurements in identical subjects, the biological specificity of the MRI-derived predictions is not fully confirmed. These spatial patterns of the target atlas are also evident in the group-level averages of both HC subjects and PD patients, though the latter show a noticeable reduction in signal intensity. The target DaT atlas introduces atlas-to-cohort differences (e.g., age, sex, and acquisition domain) that could attenuate effect sizes.

PD diagnosis in this cohort was based on specialist clinical assessment and was not systematically confirmed by paired dopaminergic imaging in all participants. Although this reflects routine clinical practice, some residual risk of diagnostic misclassification cannot be excluded. DaT prediction would likely benefit from a larger dataset of paired radiotracer scans and MRI measurements in HC subjects and PD patients. Non-averaged individual data might be able to avoid biological inaccuracies in averaged atlas data and include PD patients in the training data. A larger sample size could further prevent memorization of subject identity and reliance on spatial artifacts during single-voxel training, which we had to address with the adversarial heads during contrastive learning. Apart from the modest sample size (HCs n=21 , PD n=10 ), the use of a basal ganglia-centered slab acquisition represents a further limitation of this work.

The present results in DaT prediction motivate a validation study across a larger cohort, with subject-wise ground truth DaT scans. The ability to predict DaT also hints at the possibility to predict the distribution of further neurochemical targets of interest from generic multiparametric MRI data.

## 6 Conclusion

A subject- and coordinate-invariant multispectral MRI embedding, coupled with a simple regressor to a normative DaT atlas, detects a significant reduction of putaminal DaT signal in PD. These results provide proof-of-concept for the feasibility of high-field multispectral MRI as a noninvasive, neurochemistry-informed biomarker for Parkinson’s disease and motivate larger, individualized studies.

## Supplementary Material

Supplementary Material

## Data Availability

The data used in this study are available from the corresponding authors upon reasonable request. The preprocessing, training, and evaluation code are available at: https://github.com/mert-o/dat_prediction.
