# Supplementary material for: Multispectral 7 Tesla MRI as a potential predictor of dopamine transporter deficiency in Parkinson’s disease
Source: Imaging Neurosci (Camb). 2026 May 26;4:IMAG.a.1241. doi: 10.1162/IMAG.a.1241 (PMC13214570; doi:10.1162/IMAG.a.1241)
Supplement: Supplementary Material [file IMAG.a.1241_supp.pdf]

Supplementary material for multispectral 7 Tesla MRI as a potential predictor of dopamine transporter deficiency in Parkinson's Disease

April 22, 2026

1 Saliency Analysis

We employed gradient-based saliency analysis to quantify the contribution of input contrasts to our pipeline's predictions. For each subject, a saliency map S was computed via backpropagation, defined as the voxel-wise absolute value of the gradient of the pipeline output y with respect to the input feature i, i.e., $S = \left| i \cdot \frac{\partial y}{\partial i} \right|$, weighted by the input magnitude to emphasize features that both strongly influence the output and exhibit substantial signal at a given voxel. To derive regional feature importance, we spatially aggregated the saliency map by computing the mean intensity within ROIs. These regional profiles were averaged across the HC cohort. The contrast importance analysis in Figure S5 revealed consistent feature contributions across regions. Unexpectedly, a dominant source of saliency arose from the z-spectrum water-peak features at both low- and high-B_1 levels, with contributions concentrated around the water resonance. Across the putamen, caudate, and striatum, the five nominal z-spectrum offsets $\{-0.6, -0.4, 0, 0.4, 0.6\}$ parts per million (ppm) accounted for approximately 41-46% of the total z-spectrum saliency at low B_1 and 42-43% at high B_1, despite representing only 5 of the 56 sampled nominal z-spectrum offsets. When considering the whole brain, these same nominal offsets explained 35-37% of the total z-spectrum saliency. In the putamen, $0\,\mathrm{ppm}$ alone accounts for approximately 14% of the total low-B_1 z-spectrum saliency, whereas the broader $\pm 0.6\,\mathrm{ppm}$ neighborhood collectively contributes approximately 41%. The shift of the saliency water peak to more negative nominal z-spectrum offsets in the caudate might be caused by lower tissue susceptibility. Outside this narrow water-centered interval, saliency decreased, with higher-offset frequencies contributing only marginally. We attributed

Patient	Age (years)	Gender	Disease duration (years)	H&Y stage	Laterality (clinical)	Tremor	Medication LED mg/day	Confirmed by DaT Scan
**PDP-1**	59	M	1.5	II	right	no	100	no
**PDP-2**	62	M	5.3	I	left	yes	415	no
**PDP-3**	56	M	1	II	left	no	None	yes
**PDP-4**	50	M	4.5	I	right	yes	None	no
**PDP-5**	71	M	3.5	II–III	left	yes	415	yes
**PDP-6**	54	M	2.5	II–IIII	right	yes	160	yes
**PDP-7**	48	M	2.5	I	right	no	605	no
**PDP-8**	73	M	0.9	I	right	yes	None	yes
**PDP-9**	51	F	1.9	I-II	right	yes	100	no
**PDP-10**	63	M	1	I	right	no	300	yes

Table S1: Overview of PD patients.

the weak sensitivity to higher CEST frequency offsets to their strong collinearity, which renders these features largely redundant for the encoder during the initial representation learning stage. QTI features showed the next highest and consistently elevated influence across regions, followed by QSM and CEST parametric maps, which contributed at a moderate but discernible level. Although individual b-tensor channels exhibit modest saliency, they collectively contribute approximately 60% of the total absolute saliency. The spatial distribution of gradient magnitudes is shown in Figure S6. The interpretation of CEST water peak saliency remains difficult and might indicate utilization of spatial information from B_0-field inhomogeneities or tissue susceptibility by the network. Future refinements of the pipeline could reduce collinearity upfront by amplifying small but biologically relevant variations in z-spectra, e.g., for offsets around NOE and Amide peaks. Future larger datasets with individualized instead of atlas-based DaT-information is required to investigate the contribution of different contrasts.

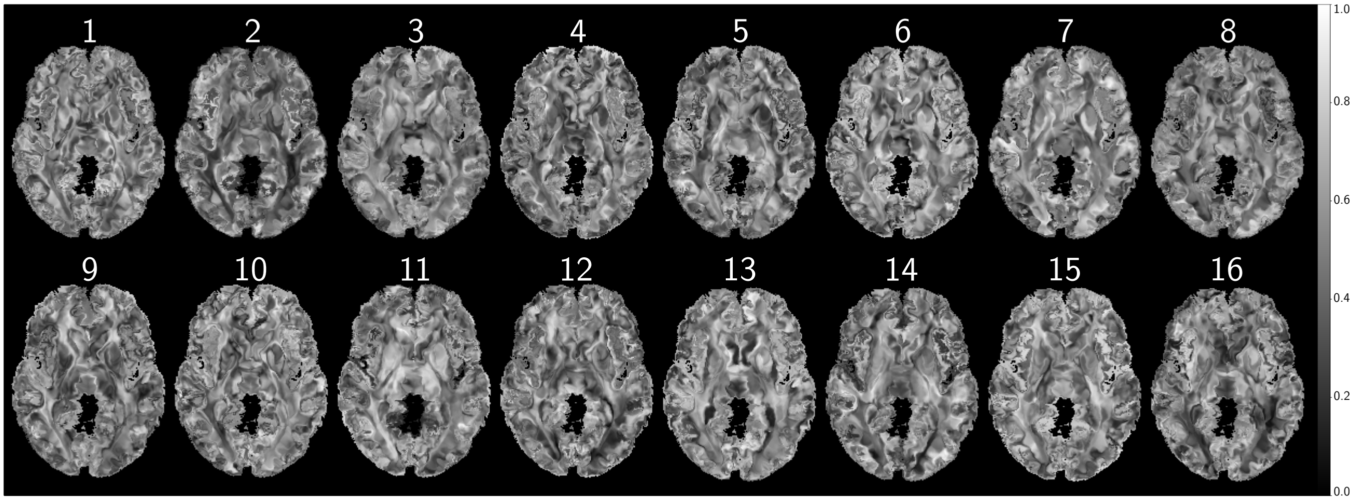

Figure S1: Example dimensions of a 16-dimensional embedding of a test HC. Numbers indicate the learned embedding dimensions.

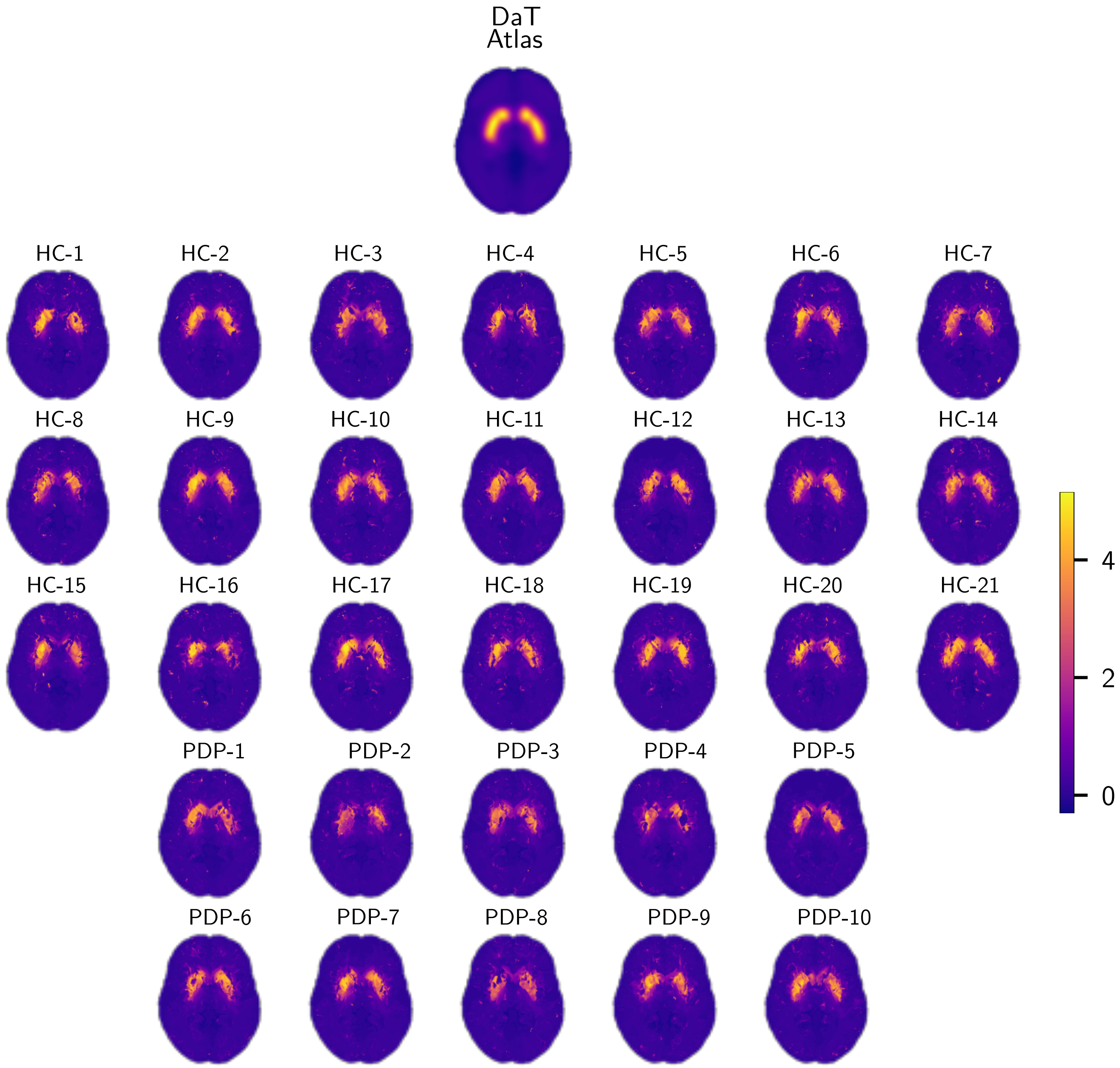

Figure S2: Individual test subject predictions of HCs and PD patients across cross-validation folds. PD patients are averaged individually across folds.

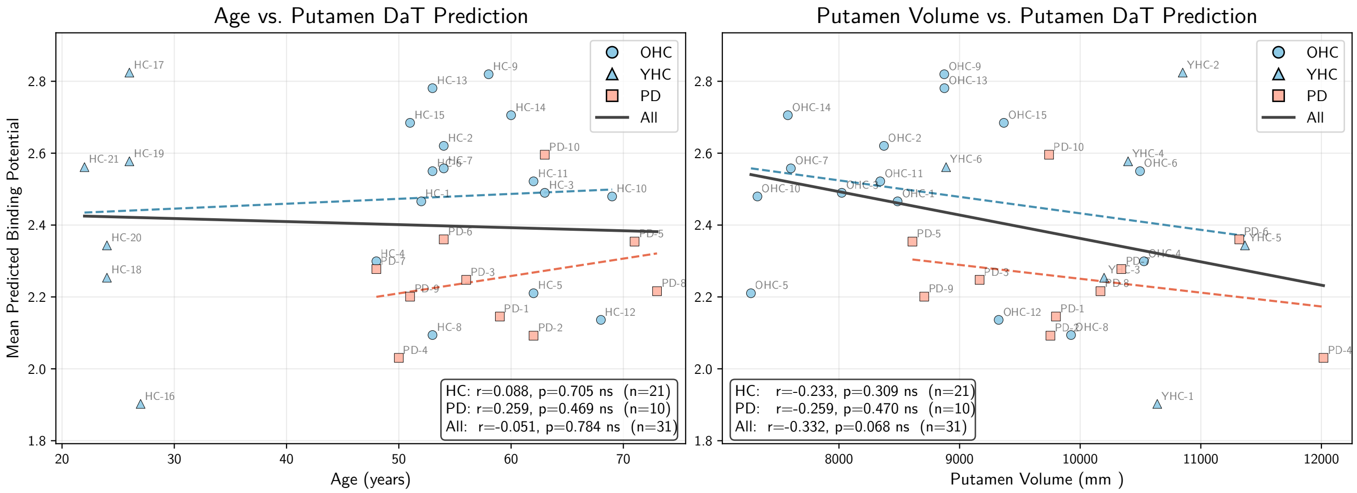

Figure S3: Relationship between predicted putaminal DaT binding potential and potential confounding variables. Left: Age vs. mean predicted putamen binding potential. Right: Putamen volume vs. mean predicted putamen binding potential. Dashed lines indicate group-wise linear fits and the solid line shows the fit across all subjects. Correlation coefficients and p-values are reported in each panel. No significant correlations were observed between predicted binding potential and either age or putamen volume, suggesting that the model predictions are not driven by age-related effects or regional atrophy.

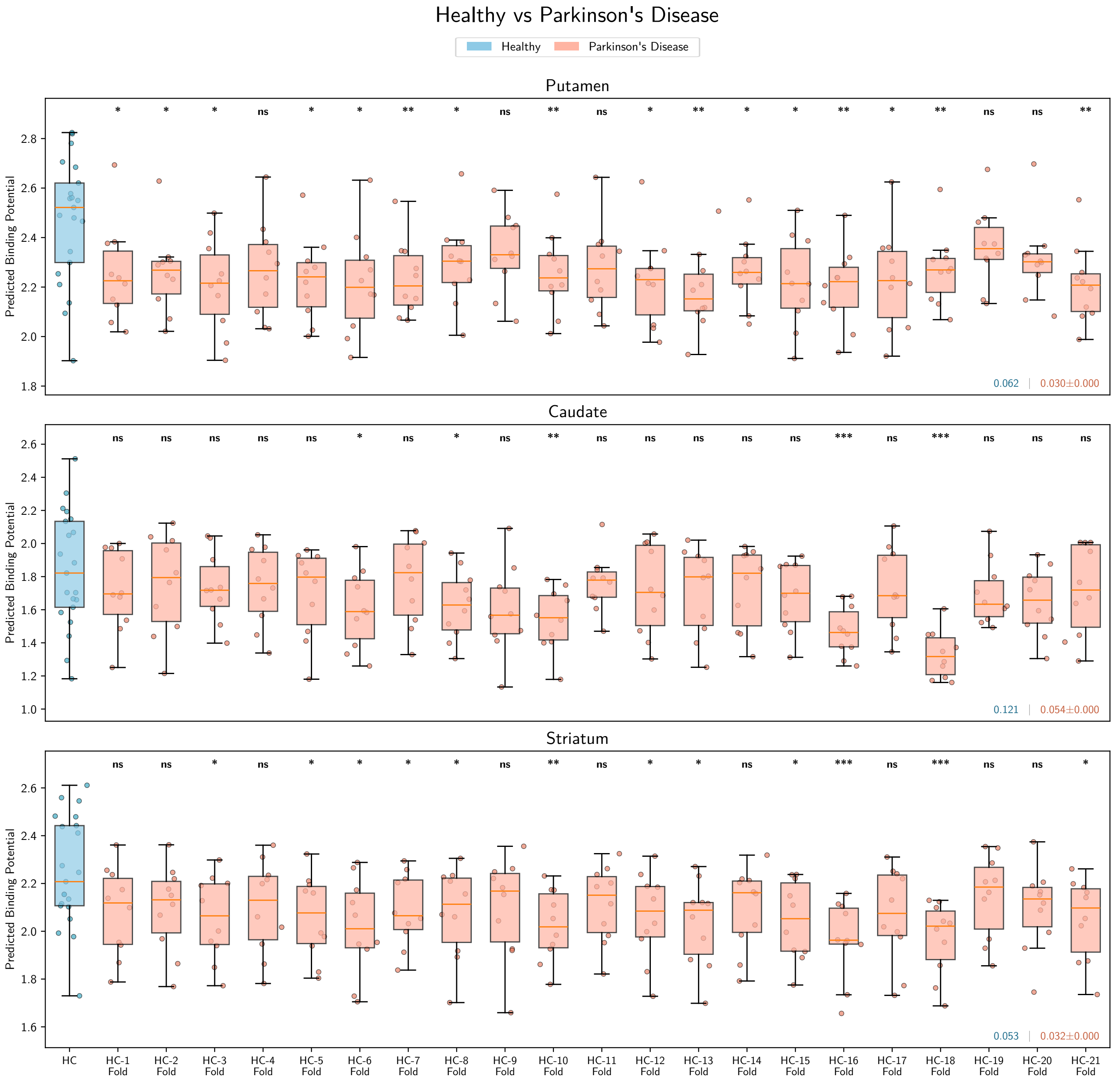

Figure S4: Individual test HC subject runs and corresponding PD predictions of that fold, compared to all test HC folds. 16 out of 21 folds show a significant difference in the putamen between HC and PD patients. *, **, and *** indicate the FDR-adjusted significance level of $q < 0.05$, $q < 0.005$, and $q < 0.0005$ respectively, whereas 'ns': not significant. Numbers in the bottom corner indicate the variance of the HC group and all folds.

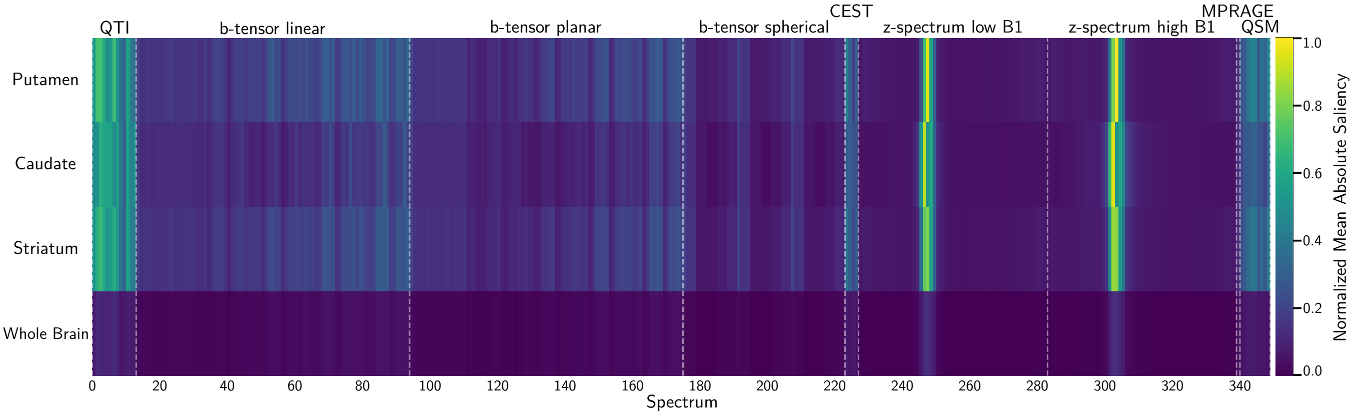

Figure S5: Mean absolute saliency values aggregated across contrasts and regions, normalized to the $[0, 1]$ range. Each column corresponds to an individual contrast feature, and each row shows the regional aggregation of voxel-wise saliency values for the putamen, caudate, striatum, and whole brain. The strongest contributions arise from the z-spectrum water-peak features at both low- and high-B_1 levels, specifically at offsets $\{-0.6, -0.4, 0, 0.4, 0.6\}$ ppm, followed by QTI contrasts. QSM and CEST parametric maps contribute moderate sensitivity, while b-tensor linear, planar, and spherical contrasts exhibit comparatively low feature-wise influence. CEST offsets far from the water resonance show minimal saliency. This hierarchy is consistent across all examined nuclei.

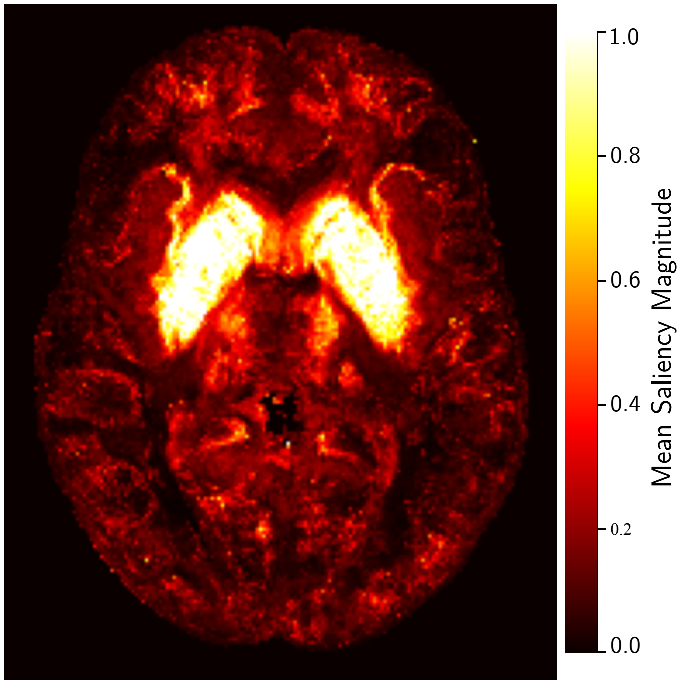

Figure S6: Spatial distribution of the saliency gradient magnitudes, normalized to the [0,1] range for visualization.
